# Soluble TREM-1 plasma concentration predicts poor outcome in COVID-19 patients

**DOI:** 10.1186/s40635-023-00532-4

**Published:** 2023-08-14

**Authors:** Sébastien Gibot, Thomas Lafon, Laurent Jacquin, Benjamin Lefevre, Antoine Kimmoun, Anne Guillaumot, Marie-Reine Losser, Marion Douplat, Laurent Argaud, Guillaume De Ciancio, Lucie Jolly, Nina Touly, Marc Derive, Catherine Malaplate, Amandine Luc, Cédric Baumann, Bruno François

**Affiliations:** 1 Médecine Intensive et Réanimation, Hôpital Central, Université de Lorraine, CHRU-Nancy, 54000 Nancy, France; 2 Emergency Department, Limoges University Hospital Center, 87000 Limoges, France; 3 Emergency Department, Hospices Civils de Lyon, Hôpital Edouard Herriot, 69003 Lyon, France; 4 Service des Maladies Infectieuses et Tropicales, Université de Lorraine, CHRU-Nancy, 54000 Nancy, France; 5 Médecine Intensive et Réanimation, Hôpital Brabois, Université de Lorraine, CHRU-Nancy, 54500 Vandoeuvre-Les-Nancy, France; 6 Département de Pneumologie, Hôpital Brabois, Université de Lorraine, CHRU-Nancy, 54500 Vandoeuvre-Les-Nancy, France; 7 Réanimation Chirurgicale, Hôpital Brabois, Université de Lorraine, CHRU-Nancy, 54500 Vandoeuvre-Les-Nancy, France; 8 Emergency Department, Hospices Civils de Lyon, Hôpital Lyon Sud Pierre Benite, 69000 Lyon, France; 9 Service de Médecine Intensive-Réanimation, Hospices Civils de Lyon, Hôpital Edouard Herriot, 69003 Lyon, France; 10 Département de Cardiologie, Hôpital Brabois, Université de Lorraine, CHRU-Nancy, 54500 Vandoeuvre-Les-Nancy, France; 11 Inotrem Sa, Faculté de Médecine de Nancy, 54500 Vandoeuvre-Les-Nancy, France; 12 Laboratoire de Biochimie, Hôpital Brabois, Université de Lorraine, CHRU-Nancy, 54500 Vandoeuvre-Les-Nancy, France; 13 Unité de Méthodologie, Data Management et Statistiques, Hôpital Brabois, Université de Lorraine, CHRU-Nancy, 54500 Vandoeuvre-Les-Nancy, France; 14 Réanimation Polyvalente et Inserm CIC-1435 & UMR-1092, CHU Limoges, 87000 Limoges, France; 15 Service de Médecine Intensive et Réanimation, Hôpital Central, 29 Avenue de Lattre de Tassigny, 54035 Nancy Cedex, France; 16 Inserm CIC 1435, Limoges University Hospital Center, 87000 Limoges, France; 17 Centre de Ressources Biologiques Lorraine, CHRU Nancy, Hôpital Brabois, 54500 Vandoeuvre-Les-Nancy, France

**Keywords:** COVID-19, ARDS, TREM-1, Prognostication

## Abstract

**Background:**

The immuno-receptor Triggering Expressed on Myeloid cells-1 (TREM-1) is activated during bacterial infectious diseases, where it amplifies the inflammatory response. Small studies suggest that TREM-1 could be involved in viral infections, including COVID-19. We here aim to decipher whether plasma concentration of the soluble form of TREM-1 (sTREM-1) could predict the outcome of hospitalized COVID-19 patients.

**Methods:**

We conducted a multicentre prospective observational study in 3 university hospitals in France. Consecutive hospitalized patients with confirmed infection with SARS-CoV-2 were enrolled. Plasma concentration of sTREM-1 was measured on admission and then at days 4, 6, 8, 14, 21, and 28 in patients admitted into an ICU (ICU cohort: ICUC) or 3 times a week for patients hospitalized in a medical ward (Conventional Cohort: ConvC). Clinical and biological data were prospectively recorded and patients were followed-up for 90 days. For medical ward patients, the outcome was deemed complicated in case of requirement of increased oxygen supply > 5 L/min, transfer to an ICU, or death. For Intensive Care Unit (ICU) patients, complicated outcome was defined by death in the ICU.

**Results:**

Plasma concentration of sTREM-1 at inclusion was higher in ICU patients (*n* = 269) than in medical ward patients (*n* = 562) (224 pg/mL (IQR 144–320) vs 147 pg/mL (76–249), *p* < 0.0001), and higher in patients with a complicated outcome in both cohorts: 178 (94–300) vs 135 pg/mL (70–220), *p* < 0.0001 in the ward patients, and 342 (288–532) vs 206 pg/mL (134–291), *p* < 0.0001 in the ICU patients. Elevated sTREM-1 baseline concentration was an independent predictor of complicated outcomes (Hazard Ratio (HR) = 1.5 (1.1–2.1), *p* = 0.02 in ward patients; HR = 3.8 (1.8–8.0), *p* = 0.0003 in ICU patients). An sTREM-1 plasma concentration of 224 pg/mL had a sensitivity of 42%, and a specificity of 76% in the ConvC for complicated outcome. In the ICUC, a 287 pg/mL cutoff had a sensitivity of 78%, and a specificity of 74% for death. The sTREM-1 concentrations increased over time in the ConvC patients with a complicated outcome (*p* = 0.017), but not in the ICUC patients.

**Conclusions:**

In COVID-19 patients, plasma concentration of sTREM-1 is an independent predictor of the outcome, although its positive and negative likelihood ratio are not good enough to guide clinical decision as a standalone marker.

**Supplementary Information:**

The online version contains supplementary material available at 10.1186/s40635-023-00532-4.

## Introduction

An impressive set of research has been published in the past 3 years that focussed on the prognostication of patients suffering from COVID-19 [1]. Many biomarkers have been investigated in this setting, from simple ones such as C-reactive protein or lymphocyte count to more sophisticated, such as interleukins or complement [2–6]. However, most of these studies were retrospective and involved a limited number of patients.

The use of a prognostic tool may help clinicians to promptly recognize patients at high risk of death for which early specific interventions should be started.

In infectious diseases, the Triggering Receptor Expressed on Myeloid cells-1 (TREM-1) pathway is drawing increased attention. TREM-1 is an immune receptor broadly expressed by several immune, epithelial, and endothelial cells whose expression becomes up-regulated in infections [7]. Once activated, TREM-1 amplifies the inflammatory response [8]. Besides its membrane-bound form, TREM-1 could be released by proteolytic cleavage [9] and measured in biological fluids (such as plasma). The soluble TREM-1 (sTREM-1) concentration constitutes a specific marker of the TREM-1 pathway activity [10]. We and others have shown that increased sTREM-1 plasma concentration is a robust predictor of death in septic patients [11, 12]. The TREM-1 pathway can be modulated by the use of nangibotide, a specific TREM-1 inhibitor, for which safety has been demonstrated in humans [13]. In phase 2a clinical trial in septic shock patients, there was a trend toward a clinical benefit provided by nangibotide, especially in patients presenting an over-activation of the TREM-1 pathway [14].

A relationship between increased sTREM-1 plasma concentration and COVID-19 severity has been suggested, though with a low number of critically ill patients admitted into an Intensive Care Unit (ICU) [15–17].

We here aim to investigate, in a prospective multicentre study, whether sTREM-1 plasma concentration could predict the outcome of hospitalized COVID-19 patients. The secondary objective was to evaluate the relationship between sTREM-1 time-course and patients outcome. We hypothesize that high sTREM-1 plasma concentrations would be associated with a complicated outcome.

## Materials and methods

### Study oversight and participants

This was a multicentre prospective observational study conducted in 3 university hospitals in France (Nancy, Limoges, and Lyon) from September 2020 to November 2021.

Consecutive patients were included if they had an RT-PCR-proven SARS-CoV-2 infection and required hospitalization either in a medical ward (‘Conventional cohort’) or in an Intensive Care Unit (ICU) (‘ICU cohort’). Inclusion was performed in the emergency room, the medical ward, or in the ICU. Patients could not be enrolled in case of pregnancy or legal protection. Seventy-two healthy blood donors at the Etablissement Français du Sang served as controls.

Written informed consent was obtained from the patient or his legal representative before enrolment. The study was conducted following the principles of the Declaration of Helsinki and the Good Clinical Practice guidelines of the International Council for Harmonization. The study was approved by Comité de Protection des Personnes Est III, and registered with the number NCT04544891 (www.clinicaltrials.gov).

### Data collection

Clinical and biological data were prospectively recorded in an electronic case report form (Cleanweb, Telemedicine Technologies, France). Patients were followed-up for 90 days for the following clinical outcomes (discharged patients were telephone called): ICU admission, respiratory support requirement, vasopressor corticosteroids, and antibiotics use, extra renal therapy needs, the incidence of thromboembolic events, the incidence of nosocomial infections, length of stay, and mortality.

### Measurements

Blood was sampled at inclusion and at days 4, 6, 8, 14, 21, and 28 in patients admitted directly into an ICU or on Mondays, Wednesdays, and Fridays for patients hospitalized in a medical ward. After centrifugation at 1500G, 20 °C for 10 min, plasma was aliquoted and stored at − 80 °C until use.

Plasma sTREM-1 concentrations were measured using an analytically validated ELISA assay according to regulatory requirements (EMA 2011) with a commercially available research use only ELISA assay (Human TREM-1 Quantikine^®^ ELISA kit, R&D Systems). Interleukin-6 plasma concentrations were measured using the automated microfluidic analyzer ELLA (BioTechne, Minneapolis, MN, USA), according to the manufacturer’s protocol. In addition, the following cytokines were also measured by ELLA in the ICU patients: Interleukins-1b, -8, -10; Chemokine ligand 2 (CCL2); Interferon-γ, and Angiopoietin-2.

### Definition of a complicated outcome

In medical ward patients, the outcome was deemed complicated in case of requirement of increased oxygen supply > 5 L/min (for those initially receiving < 5 L/min) or high flow oxygen therapy (HFOT) (for those initially not under HFOT), transfer to an ICU, or death, whichever comes first.

In ICU patients, a complicated outcome was defined by death in the ICU.

The main objective of this study was to determine if baseline plasma concentration of sTREM-1 could predict a complicated outcome. The secondary objective was to evaluate the relationship between sTREM-1 time-course and patients outcome.

### Statistical analysis

We hypothesized that 40% of patients with elevated baseline sTREM-1 would present a complicated outcome vs 25% of patients with low sTREM-1. With a alpha risk set at 5% and a power of 90%, and assuming 20% of missing data or lost to follow up, 804 subjects were deemed necessary.

The categorical variables were described by the number and percentage of their modalities, and the continuous variables by mean ± standard deviation or median and quartiles (depending on the nature of the distribution of the variable).

The effect of TREM-1 activation via the measurement of sTREM-1 (and that of the daily variation of sTREM-1) on the complicated outcome of patients was studied by a survival analysis using a Cox model. We calculated the daily variation of sTREM-1 as ((Time x value-baseline value)/baseline value) × 100/number of days between baseline and time x. The tested association was adjusted for variables significantly associated with the 2 parameters of interest studied (sTREM-1 concentration and complicated outcome). The strength of association was estimated by a hazard ratio and its 95% confidence interval.

An optimal threshold for sTREM-1 concentration was determined by calculation of the Youden index for sTREM-1 based upon ROC curves and by the median value of the daily change in sTREM-1. Using these optimal cutoffs, Kaplan–Meier curves were constructed and compared with a Log-Rank test, and sensitivity, specificity, positive and negative predictive values were calculated. The changes of sTREM-1 plasma concentrations over time were analyzed using a repeated measures mixed model.

The significance level was set at 5% for all statistics performed. Analyses were performed using SAS v9.4 (SAS Institute, Cary NC, USA).

## Results

### Characteristics of the conventional and ICU cohorts

**Fig. 1:**
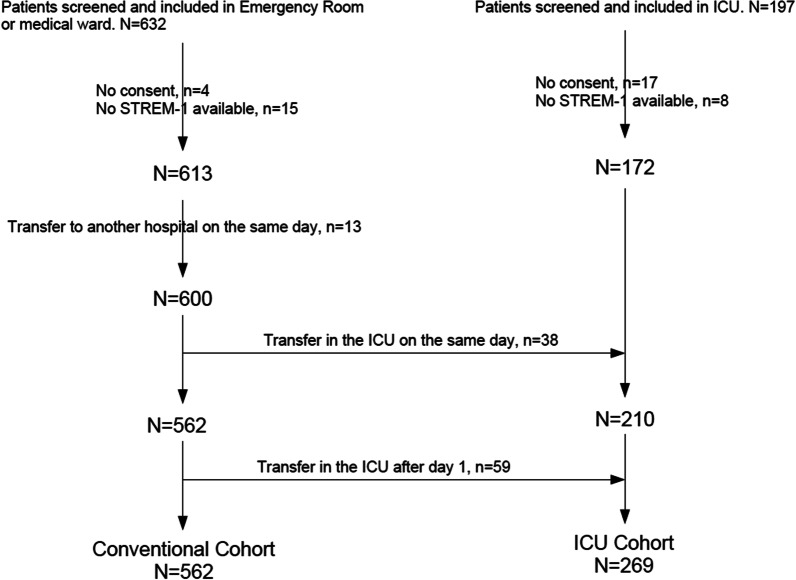
Flow chart of the study

From September 2020 to November 2021, 829 patients with a proven SARS-CoV-2 infection were screened for inclusion. Out of them, 57 were not included because of lack of informed consent, no blood sampling for sTREM-1 measurement, or immediate transfer to a non-participating hospital. Among the 600 patients included in the medical ward or emergency room, 38 were transferred to the Intensive Care Unit (ICU) the same day, leaving 562 patients in the Conventional cohort (ConvC). Fifty-nine patients were secondary admitted into the ICU: the ICU cohort (ICUC), therefore, comprised 269 patients (Fig. 1).

The mean age was 61 years. Most of the patients were male, overweighted, and suffered from comorbidities (mainly arterial hypertension and diabetes). Immunosuppression was uncommon (Table 1).

**Table 1:** Baseline characteristics of the patients

Characteristic*	Conventional cohort (*n* = 562)	ICU cohort (*n* = 269)
Age, y	61.1 ± 15.1	61.2 ± 11.5
Sex, male, *n* (%)	349 (62.1)	187 (69.5)
Body mass index, kg/m^2^	29.0 ± 5.8	30.9 ± 5.6
Previous medical history, %		
Smokers	35.1	38.8
Alcohol	19.3	12.8
Arterial hypertension	42.5	49.8
Ischemic cardiopathy	7.3	7.5
Pulmonary embolism	3.9	3.4
Deep vein thrombosis	4.8	3.4
Diabetes	23	33.5
COPD	6.2	3.3
Asthma	8.50	5.6
Obstructive sleep apnea	10	8.6
Cancer	10.7	7.8
Immunosuppression	3.7	4.1
Daily treatments, %		
Inhibitors of angiotensin converting enzyme	15.2	15.6
Aldosterone antagonists	15.7	16.4
Beta-blockers	18.4	19.3
Corticosteroids	8.4	7.1
Immunosuppressors	2.7	3
Antiplatelets agents	14.4	11.9
Anticoagulants	11.4	8.1
COVID-19 symptoms, %		
Fever	69.4	65
Asthenia	73.3	59
Cough	72.2	62.9
Dyspnea	70.3	81.6
Chest pain	20.8	11
Myalgia	35.5	25.5
Diarrhoea	36.1	27.9
Vomiting	22.4	14.7
Headache	30.6	17.2
Cutaneous rash	2.8	2.7
Ageusia	24.6	13.7
Anosmia	20.7	13.7
pH	7.50 (7.40–7.50)	7.50 (7.40–7.50)
PaO_2_, mmHg	71.1 (62.3–84)	72.9 (61.6–88.2)
PaCO_2_, mmHg	34.5 (31.2–37.5)	34.0 (30.0–39.1)
PaO_2_/FiO_2_	286 (188–331)	102.0 (80.5–155.5)
Supplemental Oxygen < 5L/min, %	91.3	
Supplemental Oxygen > 5L/min, %	5.1	
High flow oxygenation, %	3.6	60.6
Invasive mechanical ventilation, %	0	30.5
Vasopressors, %	0	10
Extra renal therapy, %	0	0.4
Antibiotics, %	20.3	37.5
sTREM-1, pg/mL	147 (76–249)	224 (144–320)
Interleukin-6, pg/mL	16 (7–36)	45 (15–98)
Interleukin-1b, pg/mL		0.2 (0.2–0.2)
Interleukin-8, pg/mL		20 (14–31)
Interleukin-10, pg/mL		13 (8–22)
Interferon-γ, pg/mL		4 (1–10)
Chemokine ligand-2, pg/mL		363 (282–691)
Angiopoietin-2, pg/mL		1298 (896–2010)

*Data are presented as means (standard deviation), percentages, or medians (interquartile range)

Regarding the respiratory support at inclusion, 91.3% were receiving < 5 L/min supplemental oxygen, 5.2% receiving > 5 L/min O_2_, and 3.6% under high-flow oxygen therapy (HFOT) in the ConvC.

In the ICU cohort, 60.6% were receiving HFOT, and 30.5% were under invasive mechanical ventilation. The use of antibiotics was frequent at inclusion: 20.3% in the ConvC, and 37.5% in the ICUC.

Interestingly, we did not find any indication of a ‘cytokine storm’ in the ICU patients as all measured cytokines remained low at inclusion (Table 1).

Among the ConvC patients, 27.4% presented with a complicated outcome after a median of 2 days (IQR 1–3): increase in oxygen requirement (15.7%), transfer to the ICU (10.1%), or death in the ward (1.6%). The hospital length of stay was of 7 days (IQR 5–10) and the mortality rate at day 90 of 8.2%. One patient remained hospitalized on day 90.

In the ICUC, the mortality rate was 18.5% at day 90, and 9 patients (3.4%) remained hospitalized with 2 of them still in the ICU. Death occurred after a median of 6 days (IQR 5–8). 55.4% of patients have been under invasive mechanical ventilation for a median of 15 days (IQR 9–27). The incidence of nosocomial infection (mainly ventilator-associated pneumonia) was as high as 42%. In both cohorts, the use of corticosteroids was common (64.8% in the ConvC, and 69.9% in the ICUC) (Table 2).

**Table 2:** Main outcomes at day 90

Outcome*	Conventional cohort (*n* = 562)	ICU cohort (*n* = 269)
Increase in Oxygen requirement, %	15.6	
Transfer to the ICU, %	10.1	
Death in the ward, %	1.6	
Death in the ICU, %		17.8
Mortality, %	8.2	18.5
Antibiotics, %	29.0	65.1
Corticosteroids, %	64.8	69.9
Vasopressors, %	3.7	37.2
Invasive mechanical ventilation (IMV), %	3.6	55.4
Duration of IMV, days	15 (9–23)	15 (9–27)
Extra renal therapy, %	1.1	7.1
Nosocomial infection, %	2.7	42.0
Thromboembolic event, %	3.0	10.1
Hospital length of stay, days	7 (5–10)	16 (10–30)

*Data are presented as percentages or medians (interquartile range)

### sTREM-1 is increased in patients with a complicated outcome

**Fig. 2:**
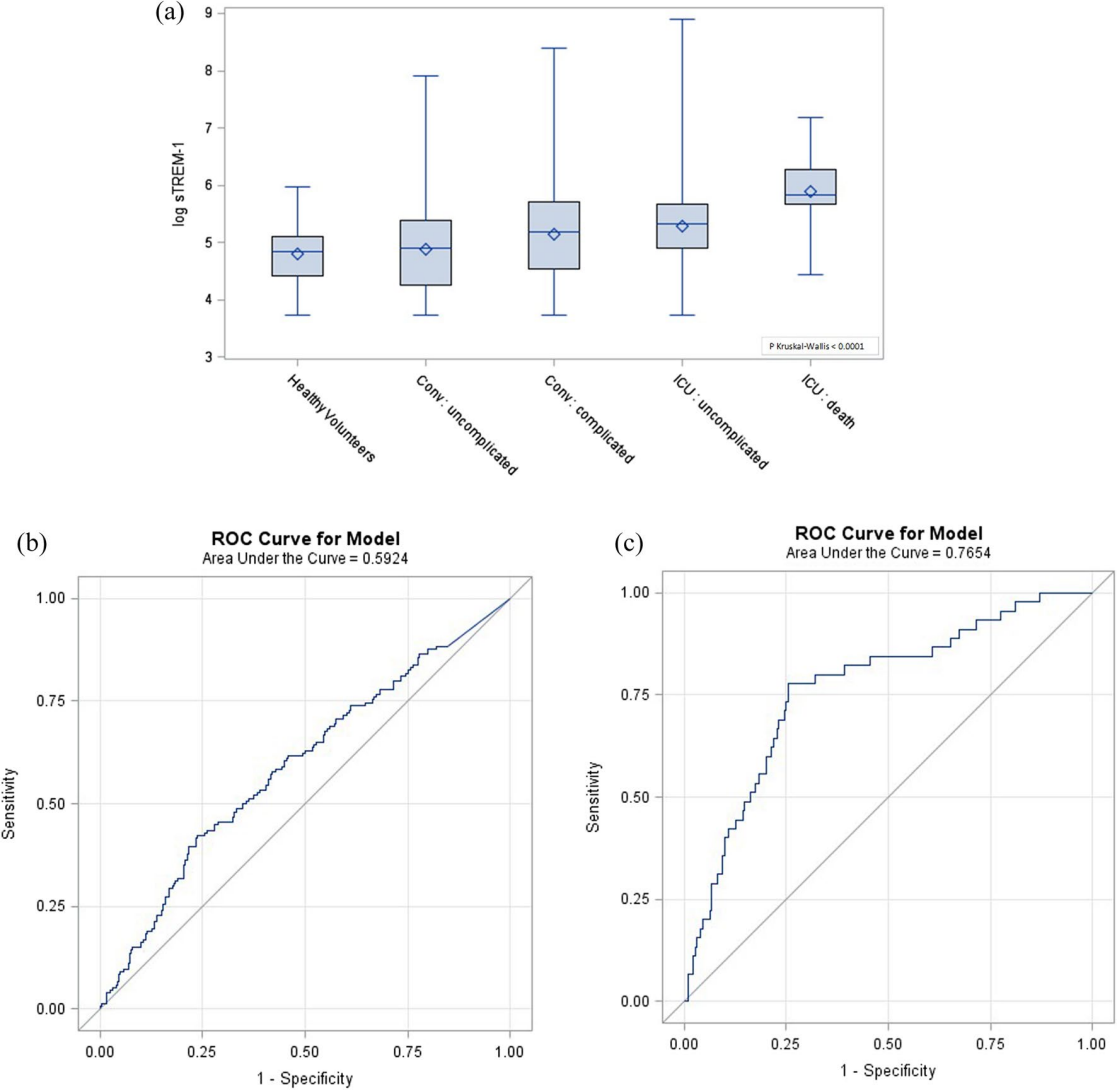
sTREM-1 concentration and its discriminatory power for complicated outcome. **a** sTREM-1 plasma concentrations at admission in the conventional cohort (medical ward) and the ICU cohort of patients according to their outcome. P values were calculated with the Mann–Whitney *U* test. *p < 0.0001. **b** Receiver-operating curves for sTREM-1 based on the outcome of the conventional cohort, and **c** the ICU cohort of patients

Plasma concentration of sTREM-1 at inclusion was higher in COVID-19 patients than in healthy volunteers [170 (94–284) vs 127 pg/mL (83–164), *p* < 0.0001], and higher in the ICU than in the ConvC patients (224 (IQR 144–320) vs 147 pg/mL (76–249), *p* < 0.0001). Soluble TREM-1 was also higher in patients with a complicated outcome in both cohorts: 178 (94–300) vs 135 pg/mL (70–220), *p* < 0.0001in the ConvC, and 342 (288–532) vs 206 pg/mL (134–291), *p* < 0.0001 in the ICUC (Fig. 2a).

**Fig. 3:**
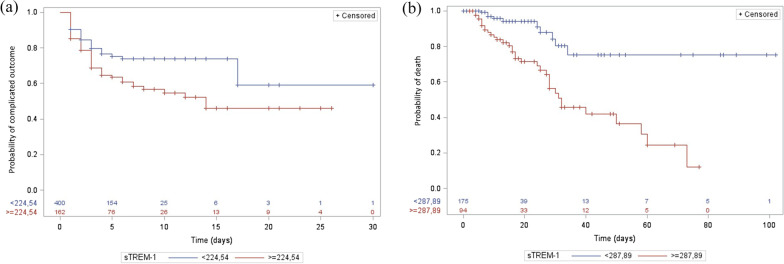
Survival curves according to baseline sTREM-1 plasma concentration. Kaplan Meier curves (**a**) in the conventional cohort for complicated outcome with sTREM-1 cutoff set at 224 pg/mL, and (**b**) in the ICU cohort for death with sTREM-1 cutoff set at 287 pg/mL. LogRank tests with respective *p* values at 0.0013 and < 0.0001

We next used the Youden index to determine the best sTREM-1 cutoffs in predicting complicated outcomes. We found that an sTREM-1 plasma concentration of 224 pg/mL had a sensitivity of 42%, a specificity of 76%, a positive predictive value of 40%, a negative predictive value of 78%, a positive likelihood ratio (LR) of 1.75, and a negative LR of 0.76 in the ConvC. The area under the ROC curve was 0.59 (95% IC, 0.54–0.65) (Fig. 2b). A complicated outcome occurred in 22.3% of patients with an sTREM-1 concentration < 224 pg/mL and in 40.1% of patients with sTREM-1 > 224 pg/mL (*p* < 0.0001). The discriminative power of sTREM-1 was further evaluated through Kaplan Meier curves and the LogRank test was of 0.0013 (Fig. 3a).

In the ICUC, a 287 pg/mL cutoff led to a sensitivity of 78%, a specificity of 74%, a positive predictive value of 37%, a negative predictive value of 94%, a positive LR of 3.0, and a negative LR of 0.3. The area under the ROC curve was 0.76 (95% IC, 0.69–0.84) (Fig. 2c). Death occurred in 5.7% of patients with a baseline sTREM-1 < 288 pg/mL and in 37.2% of patients with sTREM-1 > 288 pg/mL (*p* < 0.0001). The Kaplan Meier curves, based upon this cutoff, are depicted in Fig. 3b and the LogRank test was < 0.0001.

### Baseline sTREM-1 is an independent predictive factor of complicated outcome

In the Conventional Cohort, patients presenting with a high baseline sTREM-1 concentration (> 224 pg/mL) were older, had more comorbidities (and associated treatments), renal function alteration, higher neutrophils count, and lower lymphocytes count, than patients with low sTREM-1 (Additional file 1: Table S1). Seventeen variables with p values < 0.1 in the univariate analysis based upon the sTREM-1 cutoff and in the bivariate Cox analysis of complicated outcome were candidates to the multivariate-adjusted Cox model: body mass index; hemoptysis, dyspnea, ageusia, anosmia, confusion; history of ischemic cardiopathy, COPD, immunosuppression; daily treatment with beta-blockers, corticosteroids, immunosuppressors, metformin; corticosteroids at inclusion; plasma concentration of urea, creatinine, sodium, and lymphocytes count. One or more data were missing for 66 patients, leaving 496 patients (88.2%) for the analysis. Only 3 variables remained significantly associated with a complicated outcome: high sTREM-1 baseline concentration (> 224 pg/mL) (Hazard Ratio (HR) = 1.5 (95% IC, 1.1–2.1), *p* = 0.02), COPD (HR = 2.0 (95% IC, 1.2–3.3), *p* = 0.004), and under corticosteroids at inclusion (HR = 2.3 (95% IC, 1.5–3.4), *p* < 0.0001) (Table 3).

**Table 3:** Cox multivariate regression model

	*N* = 496	Complicated outcome in ward	Hazard ratio	*p* value
Baseline sTREM-1 cutoff				
< 224 pg/mL	351	83 (23.6%)	1	
≥ 224 pg/mL	145	60 (41.4%)	1.5 (1.1–2.1)	0.0216
COPD				
No	464	124 (26.7%)	1	
Yes	32	19 (59.4%)	2.0 (1.2–3.3)	0.0045
Corticosteroids at inclusion				
No	191	31 (16.2%)	1	
Yes	305	112 (36.7%)	2.3 (1.5–3.4)	< 0.0001

	*N* = 247	Death in ICU	Hazard ratio	*p* value
Baseline sTREM-1 cutoff				
< 287 pg/mL	160	10 (6.3%)	1	
≥ 287 pg/mL	87	34 (39.1%)	3.8 (1.8–8.0)	0.0003
Ischemic cardiopathy				
No	229	34 (14.8%)	1	
Yes	18	10 (55.6%)	2.9 (1.4–6.1)	0.004
Chronic treatment with Corticosteroids				
No	230	36 (15.7%)	1	
Yes	17	8 (47.1%)	2.9 (1.3–6.7)	0.01

In the ICU cohort, a similar pattern of differences as in the ConvC was observed between patients with elevated sTREM-1 (> 288 pg/mL) or not with older age, and more comorbidities (Additional file 1: Table S2). Again, 17 variables were deemed candidates for the multivariate-adjusted Cox model. One or more data were missing for 22 patients leaving 247 (92%) for the analysis. Only 3 variables remained significantly associated with death: high sTREM-1 (HR = 3.8 (95% IC, 1.8–8.0), *p* = 0.0003), history of ischemic cardiopathy (HR = 2.9 (95% IC, 1.4–6.1), *p* = 0.004), and daily chronic treatment with corticosteroids (HR = 2.9 (95% IC, 1.3–6.7), *p* = 0.01) (Table 3).

### Time-course of sTREM-1

The sTREM-1 concentrations increased over time in the ConvC patients with a complicated outcome (*p* = 0.017) (Additional file 1: Figure S1), and the daily variation of sTREM-1 was significantly associated with a complicated outcome [adjusted hazard ratio 1.76 (IQR 1.03–3.00)]. By contrast, the daily variation of sTREM-1 was not associated with death in the ICU [adjusted hazard ratio 1.70 (IQR 0.94–3.06)]: this is in line with the fact that sTREM-1 did not change significantly over time in ICU (Additional file 1: Figure S1).

**Fig. 4:**
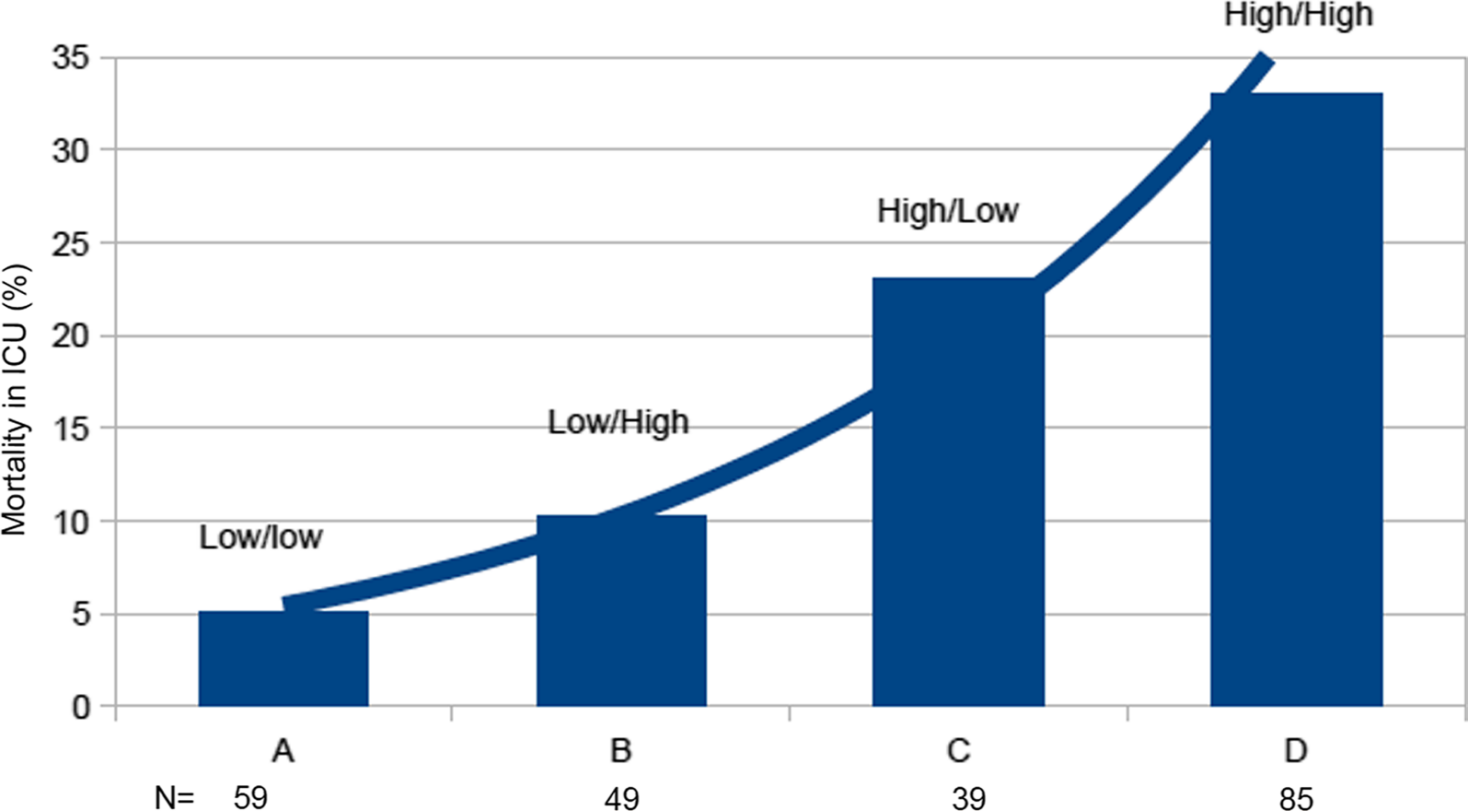
Mortality in ICU according to sTREM-1 kinetics. ICU patients were segregated into four different groups according to the sTREM-1 baseline level and kinetics. Group A: patients with low baseline sTREM-1 (< median: 224 pg/mL) that remained low (*n* = 59); Group B: low sTREM-1 that increased by more than 20% (*n* = 49); Group C: high sTREM-1 (> 224 pg/mL) that decreased of more than 20% (*n* = 39); and Group D: high sTREM-1 that stayed high (*n* = 85). The associated mortality rates were, respectively, 5.1, 10.2, 23.1, and 33% in these different groups. *p* values were obtained with Chi-2 test. *p < 0.0001

Finally, we sought to evaluate the trajectory of sTREM-1 in the ICU. In 232 patients, a second sTREM-1 measurement was obtained 4 days after admission. Four different trajectories could be observed: low baseline sTREM-1 (< median: 224 pg/mL) that remained low (*n* = 59); low sTREM-1 that increased by more than 20% (*n* = 49); high sTREM-1 (> 224 pg/mL) that decreased of more than 20% (*n* = 39); high sTREM-1 that stayed high (*n* = 85). The associated mortality rates were, respectively, 5.1, 10.2, 23.1, and 33% in these different groups (Fig. 4).

## Discussion

In this large multicentre prospective study, we confirmed that sTREM-1 plasma concentrations are increased in COVID-19 patients and that sTREM-1 elevation is an independent predictor of complicated outcomes both in the ward and in the ICU, although its positive and negative likelihood ratio are not good enough to guide clinical decision as a standalone marker. However, in the ConvC patients the daily variation of sTREM-1 was significantly associated with a complicated outcome and sTREM-1 concentrations increased over time.

Soluble TREM-1 witnesses the TREM-1 pathway activation [10]. Although this pathway has been extensively studied in bacterial infections [7, 8], its involvement during viral diseases has retained less attention.

Indeed, TREM-1 is upregulated and activated, especially in monocytes and neutrophils, after the engagement of a Toll-Like Receptor (TLR) or a Nod-Like Receptor (NLR) [18]. Viruses trigger an immune response mainly through TLR-3, -7, or -9. Mohamadzadeh et al. were the first to show that filoviruses can activate TREM-1 [19]. Since this seminal publication, other viruses have been shown to trigger the TREM-1 pathway: dengue virus, West Nile virus, enteroviruses, HBV, HCV, and HIV-1 [20–25]. Following stimulation, TREM-1 dimerizes at the membrane, associates with this accessory protein DAP-12, and then activates downstream signaling molecules including PI3K, ERK1/2, and MAP kinases that control NF-kB activation, and lead to intracellular calcium mobilization, radical oxygen species production, and cytokines/chemokines secretion [26]. The best-known function of TREM-1 is thus to amplify the inflammatory reaction. A soluble form of TREM-1 is released from the membrane after a metalloprotease-mediated proteolytic cleavage and may serve as a biomarker for the TREM-1 pathway activity [10].

We observed increased plasma concentrations of sTREM-1 both in the medical ward (Conventional cohort patients, ConvC), and in the critically ill COVID-19 patients (Intensive Care Unit cohort, ICUC) as compared to healthy volunteers. This confirmed that the TREM-1 pathway is activated during SARS-CoV-2 infection. However, sTREM-1 concentrations are lower in this population, even in the ICU patients, than during septic shock, where median level was 433 pg/mL with the same analytic method [12]. This suggests that the inflammatory response during COVID-19 is not as explosive as initially thought [27]. The low concentrations of various cytokines/chemokines we found in our ICU patients also support this fact (Table 1).

We next dichotomized patients according to their outcome: in the ConvC we defined a complicated outcome as the need for increasing oxygen support, transfer into the ICU, or death; in the ICUC complicated outcome was defined as death. Baseline sTREM-1 concentrations were higher in patients with complicated outcomes. After the determination of the best sTREM-1 cutoffs in predicting complicated outcomes, we found areas under the receiving operating curves (AUROC) of 0.59 and 0.76 in the ConvC and the ICUC, respectively. These data are in line with the 3 previously published reports. In 76 COVID-19 patients, van Singer calculated an AUROC of 0.86 in predicting intubation or death. However, the number of events was low (*n* = 17) [15]. In the 2 larger studies from de Nooijers et al. and da Silva Neto et al. (315 and 188 patients included, respectively), AUROC were 0.73 and 0.75 in predicting death [16, 17].

As expected, the clinical or biological characteristics of the patients differed according to their sTREM-1 concentrations (Additional file 1: Tables). It is interesting to note that most of these differences were similar in the 2 cohorts: patients with elevated sTREM-1 were older, had more co-morbidities and associated treatments, renal function alteration, higher neutrophil, and lower lymphocyte counts, suggesting similar profiles of severity whatever the location of the patient.

However, after adjustment, Cox model analysis revealed that elevated sTREM-1 was an independent predictor of complicated outcomes with Hazard Ratios (HR) of 1.5 and 3.8 in the ConvC and the ICUC, respectively. In the ConvC, only 2 other factors were independently associated with the outcome: history of COPD, and the use of corticosteroids at inclusion. This last factor may be counterintuitive as the RECOVERY trial showed a survival benefit of dexamethasone in hospitalized COVID-19 patients, especially those receiving supplemental oxygen [28]. Our discordant finding may stem from our composite definition of complicated outcome in the ConvC (increase oxygen support, transfer into the ICU, or death), in which death was marginal (1.6%). Therefore, corticosteroids may prevent death but not for increased oxygen requirement or need for ICU admission.

In the ICU cohort, besides sTREM-1 levels, 2 other factors were independently associated with death, though with lower HRs: history of ischemic cardiopathy, and daily chronic use of corticosteroids, reflecting the frailty of these patients.

However, even sTREM-1 is independently associated with a complicated outcome, its positive and negative likelihood ratio are not good enough to guide clinical decision as a standalone marker.

In evaluating the usefulness of a biomarker, its time-course may be of interest. When we repeat sTREM-1 measurement after 3 ± 1 days in the ICU patients, we observed 4 different trajectories: those patients with a low sTREM-1 that remained low had the lowest mortality rate (5.1%). Mortality doubled (10.2%) in patients with a low baseline value that increased by more than 20%. When sTREM-1 was initially elevated and stayed high, death occurred in 33%, whereas it dropped to 23.1% in patients in whom initially high sTREM-1 decreased by more than 20% (Fig. 4). In addition to the initial measurement, a repeat determination of sTREM-1 after a few days may help to appreciate the trajectory of the patient.

A targeted therapy (nangibotide) addressing the TREM-1 pathway is under clinical development. In a phase 2a randomized controlled trial, nangibotide showed a trend toward a clinical benefit in septic shock patients, especially those presented with an activated TREM-1 pathway (i.e., high concentrations of plasma sTREM-1) [14]. Two phase 2b trials have just been completed supporting these findings one in septic shock and one in severe COVID-19 patients (NCT04055909 and NCT04429334). In addition to serve as a prognostic biomarker, sTREM-1 could be used for patient selection, and thus population enrichment, in the next nangibotide phase 3 trials.

The main limitation of our pragmatic study is that we did not record the duration of symptoms before admission to the hospital, and therefore, we could not evaluate the relationship between sTREM-1 and the rapidity of disease progression. Another limitation is that we used a composite definition for 'complicated outcome' in the conventional cohort, and because of the very low mortality rate in these patients, it was not possible to specifically investigate the usefulness of sTREM-1 in predicting death in this population. Moreover, the choice of > 5 L/min oxygen supply (roughly corresponding to an FiO_2_ > 40%) was arbitrary decided. How a different cutoff would have altered our results is unknown. Finally, as inclusion was mostly performed during the first waves of COVID-19, we cannot extrapolate this results to more recent SARS-CoV-2 variants.

However, several strengths deserve mention including the multicentre and prospective design, a high number of patients, and a period that covered different pandemic waves (and thus different SARS-CoV-2 variants).

This study demonstrates that the TREM-1 pathway is activated in COVID-19 patients and that its magnitude of activation, appreciated by the measurement of plasma sTREM-1 concentration, is an important driver of the outcome. It also suggests that sTREM-1 could be an enrichment biomarker in upcoming studies aiming to evaluate the interest of an anti-TREM-1 approach in these patients.

## Supplementary Information

**Additional file 1. Supplementary Table 1:** Baseline characteristics according to soluble TREM-1 concentration in the conventional cohort patients. **Supplementary Table 2:** Baseline characteristics according to soluble TREM-1 concentration in the ICU cohort patients. **Supplementary Figure 1: **Plasma sTREM-1 concentration evolution according to outcome. In the Conventional cohort (left panel), sTREM-1 measurements were repeated on Mondays, Wednesdays, and Fridays; in the ICU patients, measurements were performed at days 4, 6, 8, 14, 21, and 28. The sTREM-1 concentration increased over time in the conventional cohort patients with a complicated outcome (*p* = 0.017) while it did not change significantly in the ICU patients.

## Data Availability

The data sets used and analyzed here are available from the corresponding author upon reasonable request.
