# Supplementary material for: Soluble TREM-1 plasma concentration predicts poor outcome in COVID-19 patients
Source: Intensive Care Med Exp. 2023 Aug 14;11:51. doi: 10.1186/s40635-023-00532-4 (PMC10423708; doi:10.1186/s40635-023-00532-4)

**Table S1**: Baseline characteristics according to soluble TREM-1 concentration in the Conventional cohort patients

| **Characteristic** | **Low sTREM-1 (<224pg/mL) (N=400)** | **High sTREM-1 (≥224pg/mL) (N=162)** | **p value** |
| --- | --- | --- | --- |
| **Age, years** | 56.9±13.7 | 71.7±13.0 | <0.0001 |
| **Sex, male, %** | 59.5 | 68.5 | 0.046 |
| **Body mass index, kg/m^2^** | 29.2±5.8 | 28.4±5.8 | 0.1 |
| **Previous medical history, %** |  |  |  |
| Smokers | 30.3 | 47.2 | 0.0005 |
| Alcohol | 16.6 | 26.1 | 0.01 |
| Hypertension | 32.5 | 67.3 | <0.0001 |
| Ischemic cardiopathy | 3.8 | 16.0 | <0.0001 |
| Pulmonary embolism | 2.3 | 8.0 | 0.001 |
| Deep vein thrombosis | 3.3 | 8.6 | 0.01 |
| Diabetes | 17.8 | 35.8 | <0.0001 |
| COPD | 4.0 | 11.7 | 0.0006 |
| Asthma | 9.0 | 7.4 | 0.55 |
| Obstructive sleep apnea | 7.8 | 15.4 | 0.01 |
| Cancer | 7.8 | 17.9 | 0.0004 |
| Immunosuppression | 3.3 | 4.9 | 0.34 |
| **Daily treatments, %** |  |  |  |
| Inhibitors of angiotensin converting enzyme | 11.8 | 23.5 | 0.0005 |
| Aldosterone antagonists | 12.5 | 23.5 | 0.0013 |
| Beta-blockers | 11.8 | 34.6 | <0.0001 |
| Corticosteroids | 6.0 | 14.2 | 0.0015 |
| Immunosuppressors | 2.0 | 4.3 | 0.15 |
| Antiplatelets agents | 8.0 | 30.2 | <0.0001 |
| Anticoagulant | 11.4 | 23.3 | 0.002 |
| **COVID-19 symptoms, %** |  |  |  |
| Fever | 74.3 | 57.4 | <0.0001 |
| Asthenia | 73.5 | 72.8 | 0.87 |
| Cough | 73.5 | 69.1 | 0.3 |
| Dyspnea | 70.3 | 70.4 | 0.98 |
| Chest pain | 25.3 | 9.9 | <0.0001 |
| Myalgia | 39.8 | 24.7 | 0.0007 |
| Diarrhoea | 37.5 | 32.7 | 0.28 |
| Vomiting | 24.5 | 17.3 | 0.06 |
| Headache | 37 | 14.8 | <0.0001 |
| Cutaneous rash | 3.5 | 1.2 | 0.17 |
| Ageusia | 27.3 | 17.9 | 0.02 |
| Anosmia | 24.1 | 12.3 | 0.0019 |
|  |  |  |  |
| **pH** | 7.5±0 | 7.4±0.1 | 0.01 |
| **PaO2, mmHg** | 74.6±21.1 | 83.1±31.6 | 0.08 |
| **PaCO2, mmHg** | 34.8±5 | 33.6±7 | 0.07 |
| **PaO2/FiO2** | 280.9±92.3 | 252.6±139.3 | 0.02 |
| **High flow oxygenation, %** | 1.5 | 8.6 | <0.0001 |
| **Antibiotics, %** | 17.5 | 22.7 | 0.01 |
| **Corticosteroids,%** | 61.3 | 63.0 | 0.7 |
| **Aspartate aminotransferase (UI/L)** | 55±41 | 56±34 | 0.19 |
| **Alanine aminotransferase (UI/L)** | 49±44 | 41±32 | 0.11 |
| **Alkaline phosphatase (UI/L)** | 78±42 | 87±71 | 0.07 |
| **Bilirubin (mg/L)** | 8.8±4.6 | 10.9±9.6 | 0.006 |
| **Urea (mmol/L)** | 5.3±1.8 | 10.4±6.9 | <0.0001 |
| **Creatinine (µmol/L)** | 70.7±20.7 | 130.1±122.4 | <0.0001 |
| **Glucose (mmol/L)** | 7.3±2.9 | 8.3±4.2 | 0.0007 |
| **Sodium (mmol/L)** | 137±3 | 136±4 | 0.05 |
| **Potassium (mmol/L)** | 4.0±0.6 | 4.2±0.6 | 0.0003 |
| **Chlore (mmol/L)** | 101±4 | 102±5 | 0.4 |
| **Calcium (mmol/L)** | 2.2±0.1 | 2.2±0.2 | 0.9 |
| **Proteins (g/L)** | 71±6 | 68±7 | 0.0002 |
| **Albumin (g/L)** | 33±4 | 31±4 | 0.004 |
| **Lactate (mmol/L)** | 1.3±0.6 | 1.5±0.7 | 0.01 |
| **C-reactive protein (mg/L)** | 83±69 | 107±72 | 0.0002 |
| **Ferritin (µg/L)** | 995±767 | 1169±1996 | 0.34 |
| **Hemoglobin (g/dL)** | 13.7±1.6 | 12.9±2.1 | <0.0001 |
| **Hematocrit (%)** | 41±5 | 39±6 | 0.0001 |
| **Platelets (G/L)** | 229±94 | 216±89 | 0.16 |
| **Leukocytes (G/L)** | 6.4±5.3 | 7.2±3.2 | 0.0006 |
| **Basophils (G/L)** | 0±0 | 0±0 | 0.75 |
| **Eosinophils (G/L)** | 0±0.1 | 0±0 | 0.78 |
| **Neutrophils (G/L)** | 4.7±4.4 | 5.7±3 | <0.0001 |
| **Lymphocytes (G/L)** | 1.5±5 | 0.8±0.5 | <0.0001 |
| **Monocytes (G/L)** | 0.4±0.3 | 0.5±0.4 | 0.02 |
| **International normalized ratio** | 1.1±0.4 | 1.6±1.2 | 0.02 |
| **D-Dimers (µ/L)** | 1083±949 | 1971±2024 | <0.0001 |

**Table S2**: Baseline characteristics according to Soluble TREM-1 concentration in the ICU cohort patients

| **Characteristic** | **Low sTREM-1 (<287pg/mL) (N=164)** | **High sTREM-1 (≥224pg/mL) (N=105)** | **p value** |
| --- | --- | --- | --- |
| **Age, years** | 57.8±11.5 | 66.5±9.4 | <0.0001 |
| **Sex, male, %** | 65.2 | 76.2 | 0.06 |
| **Body mass index, kg/m^2^** | 31.4±5.3 | 30.2±5.9 | 0.08 |
| **Previous medical history, %** |  |  |  |
| Smokers | 36.7 | 42.6 | 0.4 |
| Alcohol | 14.6 | 9.5 | 0.28 |
| Hypertension | 44.5 | 58.1 | 0.03 |
| Ischemic cardiopathy | 3.7 | 13.5 | 0.0029 |
| Pulmonary embolism | 2.4 | 4.8 | 0.31 |
| Deep vein thrombosis | 3.0 | 3.8 | 0.73 |
| Diabetes | 28.7 | 41.0 | 0.04 |
| COPD | 2.4 | 4.8 | 0.31 |
| Asthma | 5.50 | 5.7 | 0.93 |
| Obstructive sleep apnea | 7.9 | 9.5 | 0.64 |
| Cancer | 4.3 | 13.3 | 0.0069 |
| Immunosuppression | 1.2 | 8.6 | 0.0042 |
| **Daily treatments, %** |  |  |  |
| Inhibitors of angiotensin converting enzyme | 13.4 | 19.0 | 0.21 |
| Aldosterone antagonists | 14.6 | 19.0 | 0.33 |
| Beta-blockers | 13.4 | 28.6 | 0.0021 |
| Corticosteroids | 3.0 | 13.3 | 0.0013 |
| Immunosuppressors | 1.2 | 5.7 | 0.059 |
| Antiplatelets agents | 9.8 | 15.2 | 0.17 |
| Anticoagulant | 6.8 | 9.7 | 0.19 |
| **COVID-19 symptoms, %** |  |  |  |
| Fever | 68.1 | 60.2 | 0.18 |
| Asthenia | 59.8 | 57.8 | 0.75 |
| Cough | 65.0 | 59.4 | 0.35 |
| Dyspnea | 77.3 | 88.5 | 0.02 |
| Chest pain | 13.6 | 6.9 | 0.09 |
| Myalgia | 29.0 | 19.8 | 0.09 |
| Diarrhoea | 26.4 | 30.4 | 0.47 |
| Vomiting | 16.6 | 11.8 | 0.28 |
| Headache | 23.6 | 6.9 | 0.0005 |
| Cutaneous rash | 3.7 | 1.0 | 0.25 |
| Ageusia | 16.7 | 8.9 | 0.07 |
| Anosmia | 16.0 | 9.9 | 0.15 |
|  |  |  |  |
| **pH** | 7.5±0.1 | 7.4±0.1 | 0.0012 |
| **PaO_2_, mmHg** | 80±32 | 80±30 | 0.71 |
| **PaCO_2_, mmHg** | 35±7 | 36±9 | 0.79 |
| **PaO_2_/FiO_2_** | 130±71 | 113±56 | 0.12 |
| **Invasive mechanical ventilation, %** | 25.1 | 40.3 |  |
| **Positive end expiratory pressure (mmHg)** | 7.5±3.8 | 9.1±3.7 | 0.02 |
| **High flow oxygenation, %** | 64 | 54.2 |  |
| **Vasopressors, %** | 6.7 | 15.2 | 0.02 |
| **Extra renal therapy, %** | 0 | 1.0 | 0.39 |
| **Antibiotics, %** | 34.1 | 42.9 | 0.15 |
| **Corticosteroids,%** | 73.2 | 47.6 | <0.0001 |
| **Aspartate aminotransferase (UI/L)** | 64±42 | 53±31 | 0.06 |
| **Alanine aminotransferase (UI/L)** | 57±55 | 49±53 | 0.14 |
| **Alkaline phosphatase (UI/L)** | 74±41 | 81±40 | 0.17 |
| **Bilirubin (mg/L)** | 10±4 | 10±5 | 0.94 |
| **Urea (mmol/L)** | 7.5±11.5 | 11.3±11.1 | <0.0001 |
| **Creatinine (µmol/L)** | 76±83 | 115±108 | <0.0001 |
| **Glucose (mmol/L)** | 8±3.1 | 8.7±3.6 | 0.27 |
| **Sodium (mmol/L)** | 138±4 | 137±4 | 0.33 |
| **Potassium (mmol/L)** | 3.9±0.4 | 4.1±0.6 | 0.09 |
| **Chlore (mmol/L)** | 104±5 | 104±5 | 0.98 |
| **Calcium (mmol/L)** | 2.1±0.2 | 2.0±0.3 | 0.0048 |
| **Proteins (g/L)** | 66±7 | 64±7 | 0.01 |
| **Albumin (g/L)** | 29±4 | 26±3 | 0.0004 |
| **Lactate (mmol/L)** | 1.4±0.7 | 1.5±1.0 | 0.13 |
| **C-reactive protein (mg/L)** | 117±73 | 152±99 | 0.06 |
| **Ferritin (µg/L)** | 1488±1035 | 2391±2059 | 0.05 |
| **Hemoglobin (g/dL)** | 15.4±2.7 | 13.4±4.3 | 0.0046 |
| **Hematocrit (%)** | 41±10 | 38±5 | 0.0049 |
| **Platelets (G/L)** | 242±97 | 232±110 | 0.52 |
| **Leukocytes (G/L)** | 8±3.2 | 9.8±5 | 0.01 |
| **Basophils (G/L)** | 0±0 | 0±0 | 0.08 |
| **Eosinophils (G/L)** | 0±0 | 0±0.1 | 0.08 |
| **Neutrophils (G/L)** | 9±14.2 | 10±11.4 | 0.01 |
| **Lymphocytes (G/L)** | 3.5±14.8 | 1.9±6.3 | 0.05 |
| **Monocytes (G/L)** | 0.6±1.7 | 0.7±1.6 | 0.86 |
| **International normalized ratio** | 1.2±0.3 | 1.3±0.5 | 0.45 |
| **D-Dimers (µ/L)** | 1604±2321 | 3972±4168 | <0.0001 |


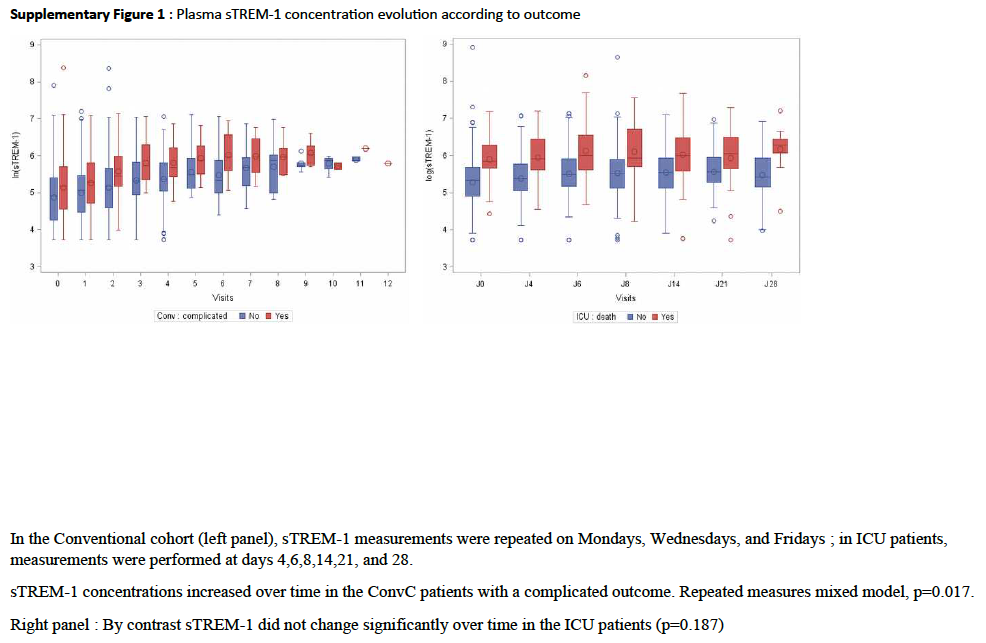

Supplement: Supplementary file 1 — Additional file 1. Supplementary Table 1: Baseline characteristics according to soluble TREM-1 concentration in the conventional cohort patients. Supplementary Table 2: Baseline characteristics according to soluble TREM-1 concentration in the ICU cohort patients. Supplementary Figure 1: Plasma sTREM-1 concentration evolution according to outcome. In the Conventional cohort (left panel), sTREM-1 measurements were repeated on Mondays, Wednesdays, and Fridays; in the ICU patients, measurements were performed at days 4, 6, 8, 14, 21, and 28. The sTREM-1 concentration increased over time in the conventional cohort patients with a complicated outcome (p = 0.017) while it did not change significantly in the ICU patients. [file 40635_2023_532_MOESM1_ESM.docx]
